# Call for a Meeting of Surgeons of the Confederate Army

**Published:** 1873

**Authors:** 


CALL FOR A MEETING OF THE SURGEONS OF THE CONFEDERATE ARMY DURING THE LATE WAR.—A circular addressed to the field and hospital surgeons of the late Confederate States, for a meeting to be held at Atlanta, Georgia, on the 20th of May, 1874, has been published in Southern and Western journals, and signed hy Dr. S. P. Moore, late Surgeon-General, and Drs. Sorrell, McGuire, Rice, Stout, Logan, Darby, and twenty other well-known Confederate surgeons; being based not alone upon the action of the Georgia Medical Association, but upon the earnest solicitations of many Confederate surgeons throughout the South. The object of the meeting is "for the advancement of Science—to rescue from oblivion all the important medical and surgical facts developed within the armies of the Confederate States during the late war."

